# CrypticIBDcheck: an R package for checking cryptic relatedness in nominally unrelated individuals

**DOI:** 10.1186/1751-0473-8-5

**Published:** 2013-02-06

**Authors:** Annick Nembot-Simo, Jinko Graham, Brad McNeney

**Affiliations:** 1 Department of Statistics and Actuarial Science, Simon Fraser University, Burnaby, Canada

**Keywords:** Cryptic relatedness, IBD estimation, Linkage disequilibrium, Gene drop simulation

## Abstract

**Background:**

In population association studies, standard methods of statistical inference assume that study subjects are independent samples. In genetic association studies, it is therefore of interest to diagnose undocumented close relationships in nominally unrelated study samples.

**Results:**

We describe the **R** package **CrypticIBDcheck** to identify pairs of closely-related subjects based on genetic marker data from single-nucleotide polymorphisms (SNPs). The package is able to accommodate SNPs in linkage disequibrium (LD), without the need to thin the markers so that they are approximately independent in the population. Sample pairs are identified by superposing their estimated identity-by-descent (IBD) coefficients on plots of IBD coefficients for pairs of simulated subjects from one of several common close relationships.

**Conclusions:**

The methods implemented in **CrypticIBDcheck** are particularly relevant to candidate-gene association studies, in which dependent SNPs cluster in a relatively small number of genes spread throughout the genome. The accommodation of LD allows the use of all available genetic data, a desirable property when working with a modest number of dependent SNPs within candidate genes. **CrypticIBDcheck** is available from the Comprehensive R Archive Network (CRAN).

## Background

It is well known that the results of genetic association studies may be confounded by the presence of undocumented relationships – a phenomenon referred to as cryptic relatedness (e.g., [1,2]). For example, [3] found that tests of association between genetic markers and quantitative phenotypes such as serum LDL tended to have inflated significance when relationships between individuals from a large Hutterite kindred were not accounted for. Before making any inference with the data, it is therefore important to understand cryptic relatedness in the study sample. To facilitate this understanding, we introduce **CrypticIBDcheck**, an **R**[4] package for exploring the presence of close relationships in a homogeneous sample of nominally unrelated individuals. Although several methods for exploring cryptic relatedness have been implemented (reviewed below), none are geared for data from candidate-gene association studies. **CrypticIBDcheck** fills this need. For ease of interpretation, the package implements exploratory displays based on popular measures of gene-identity by descent. However, a unique feature of these displays is that they accommodate population linkage disequilibrium (LD) amongst genetic markers. The accommodation of LD allows the use of data on all available markers rather than on a subset whose alleles are approximately independent in the population. This feature is attractive in candidate-gene association studies, where markers within genes are in LD but the number of genes is too small to select an independent subset of markers that is informative for relationship. Using the simulated data set analyzed in the **Examples** section we have found it possible to distinguish parent-offspring or full sibling pairs from unrelated individuals using as few as 60 candidate genes (average of five SNPs per gene). We return to the issue of how many SNPs are appropriate for analysis with **CrypticIBDcheck** in the **Conclusions** section.

The relatedness between two individuals may be defined in terms of the proportion of loci at which they share zero, one or two alleles that are identical-by-descent (IBD). We refer to these proportions as the actual IBD-sharing coefficients, or IBD coefficients. The alleles from two individuals are IBD if they are descended from a common ancestor in a given reference population (e.g., [5]). Though alleles from each of two individuals may match or be identical-by-state (IBS), they are not necessarly IBD.

**CrypticIBDcheck** uses estimated IBD coefficients to summarize possible relationships among pairs of study subjects. The approach is exploratory and graphically-based, similar to the GRR approach of [6] and the approach of [7] implemented in the ibdPlot() function of the **GWASToolsBioconductor** package.

GRR calculates and displays the mean and variance of IBS allele sharing over polymorphic loci for each pair of individuals. Pairs of known relationships form reference clusters on the plot, allowing the user to identify errors in reported relationships. In association studies of nominally unrelated individuals, however, there are no reference clusters available. In principle, reference clusters could be obtained theoretically from the joint distribution of the IBS mean and variance estimators, but it is unclear how to derive this distribution in the presence of LD.

The ibdPlot() function in **GWASTools** may be applied to view estimated IBD coefficients along with reference clusters for the unobserved, true IBD coefficients based on theoretical moments of their distribution [8]. However, in candidate-gene studies with a modest number of single-nucleotide polymorphisms (SNPs), errors introduced by estimation of IBD coefficients cannot be ignored. Hence, reference distributions for the true IBD coefficients do not adequately represent those for estimated IBD coefficents.

The idea behind **CrypticIBDcheck** is to identify closely-related study pairs by displaying their estimated IBD coefficients together with those from *simulated pairs* of known relationships. The simulated reference pairs provide an empirical joint distribution of the IBD estimators under selected relationships which, in turn, suggest possible relationships amongst study pairs. Working with simulated pairs from known relationships avoids having to derive the joint distribution of the IBD estimators when the genetic markers are in LD. Simulated pairs are obtained by gene drop on a relationship-specific pedigree, with pedigree-founder haplotypes drawn from a fitted haplotype model that accounts for LD [9]. We have implemented simulation of the following common relationships: monozygotic twins/sample duplicates, parent-offspring, full siblings, second degree (i.e., half siblings, avuncular or grandparent-grandchild) and first cousins. However, users may also specify their own custom relationships (see the **Examples** section).

The paper is structured as follows. In the **Methods** section we describe the IBD estimators and methods for gene drop simulation in the presence of LD to obtain reference clusters. The **Results and Discussion** section describes implementation details provides two examples of how to use the package. The paper ends with a **Conclusions** section that includes ideas for future work.

## Methods

### IBD estimation

There are two common approaches to estimating IBD coefficients: maximum likelihood [10-12] and the method of moments [13-15]. Typically, maximum-likelihood estimators (MLEs) are more biased than method-of-moments estimators (MMEs), especially when the number of loci is small; they are also more computationally expensive [14]. However, MMEs are less precise than MLEs and can fall outside the biologically meaningful parameter space [11].

In this section, we review a popular method of moments approach to estimating IBD coefficients introduced by [15] and implemented in PLINK. This approach assumes that the individuals are from the same homogeneous, random-mating and non-inbred population. Alleles from two individuals are considered to be IBD if they are descended from a common ancestor in some base population that we take to be relatively recent. All alleles in this base population are defined to be non-IBD. Given a SNP with alleles *A* and *a*, a pair of individuals that are, say, *AA* and *aa*, respectively, will be denoted (*AA, aa*).

Identity-by-state (IBS) for a pair of subjects is denoted by the random variable *I* and identity-by-descent by the random variable *Z*, with possible states being 0, 1, and 2 for both random variables. The IBD coefficients to be estimated are the proportions of genome shared IBD, denoted by *P*(*Z* = 0), *P*(*Z* = 1), and *P*(*Z* = 2). For a given SNP *m*, the procedure begins by expressing the prior probability of IBS sharing as

(1) $$\begin{array}{lcr} P_{m}(I=i)=\overset{i}{\underset{z=0}{\sum }}P_{m}(I=i|Z=z)P(Z=z). & & \end{array}$$

*P*(*Z* = *z*) and *P*_*m*_(*I* = *i*) are specific to the pair of subjects being considered, while the conditional SNP-specific IBS probabilities *P*_*m*_(*I* = *i*|*Z* = *z*) apply to all pairs. For a given pair of individuals at a given SNP, the above equation specifies three identities for the IBS states 0, 1, and 2. These three identities are summed over SNPs and then rearranged to express *P*(*Z* = 0), *P*(*Z* = 1), and *P*(*Z* = 2) for the pair in terms of marginal and conditional IBS probabilities. For example, in the case of *i* = *z* = 0, we obtain

$$P(Z=0)=\underset{m}{\sum }P_{m}(I=0)/\underset{m}{\sum }P_{m}(I=0|Z=0).$$

The method-of-moments estimators of IBD coefficients for a given pair are obtained by substituting estimators of the conditional SNP-specific IBS probabilities, *P*_*m*_(*I* = *i*|*Z* = *z*), pertaining to any pair and the pair’s marginal SNP-specific IBS probabilities, *P*_*m*_(*I* = *i*), into the identities and then solving for *P*(*Z* = *i*).

The marginal SNP-specific IBS probabilities, *P*_*m*_(*I* = *i*) for a pair of subjects may be estimated by the indicator function for whether the pair has *I* = *i* at the SNP. An unbiased estimator of $\underset{m}{\sum }{\hat{P}}_{m}(I=i)$ is therefore the count of SNPs at which the pair shares *i* alleles IBS. Estimates of the SNP-specific conditional IBS probabilities, *P*_*m*_(*I* = *i*|*Z* = *z*), are based on data from all subjects in the sample. Derivation of unbiased estimators of *P*_*m*_(*I* = *i*|*Z* = *z*) is more involved. To simplify notation, we temporarily drop the SNP subscript *m*. If *p* and *q* = 1−*p* denote the frequencies of *A* and *a* in the base population, then *P*(*I* = *i*|*Z* = *z*) is a function of *p* and *q*. For example, two individuals share 0 alleles IBS if they are either (*AA, aa*) or (*AA, aa*). Given that *Z* = 0, the probabilities of these genotypes are *p*^2^*q*^2^ and *q*^2^*p*^2^, respectively, leading to *P*(*I* = 0|*Z* = 0) = 2*p*^2^*q*^2^. The plug-in estimators of conditional IBS probabilities, such as *P*(*I* = 0|*Z*=0), obtained by inserting estimators $\hat{p}$ and $\hat{q}$ are biased [Additional file 1]. Unbiased estimators, expressed as the plug-in estimator multiplied by a correction factor, may be derived as described next.

Let *X* and *Y* be the counts of the alleles *A* and *a*, respectively, so that the allele frequency estimators are $\hat{p}=X/T$ and $\hat{q}=Y/T$, where *T* is twice the number of observed genotypes in the population random sample. The estimators of the conditional IBS probabilities *P*(*I* = *i*|*Z* = *z*) may be motivated by the following model. The genotype of each individual in the present population is obtained from two independent draws from an infinite base population of alleles. Consequently, the *T* alleles of a population random sample of study subjects can be viewed as a random sample from the base population. Moreover, conditional on IBD status, any pair of individuals in the present population can be viewed as independent allelic draws from the base population, with the number of draws determined by their IBD status.

For example, in the case of *Z* = 0, a random pair of individuals results from randomly drawing two pairs of alleles from the base population. An indicator variable of whether this sampling process results in *I* = 0 is an unbiased estimator of *P*(*I* = 0|*Z* = 0). An unbiased estimator is therefore the average of these indicator variables over all possible draws from the *T* alleles on which we have data; i.e., the proportion of pairs of allelic pairs with *I* = 0. The proportion can be computed as follows. The number of ways of selecting four distinct alleles from a total of *T* is *T*(*T* − 1)(*T* − 2)(*T* − 3). Without loss of generality, suppose the first two alleles are assigned to the first individual in a pair and the last two alleles to the second individual. Then the number of pairs that are (*AA, aa*) and (*AA, aa*) are *X*(*X* − 1)*Y*(*Y* − 1) and *Y*(*Y* − 1)*X*(*X* − 1), respectively. Hence,

(2) $$\begin{array}{lcr} \hat{P}(I=0|Z=0)=\frac{2X(X-1)Y(Y-1)}{T(T-1)(T-2)(T-3)}, & & \end{array}$$

is an unbiased estimator of *P*(*I*=0|*Z*=0) (see Additional file 1 for verification by direct computation). After algebra, the unbiased estimator may be expressed in terms of the allele frequency estimators and a correction factor as:

$$\begin{array}{lll} \hat{P}(I=0|Z=0)=2{\hat{p}}^{2}{\hat{q}}^{2} & \left(\frac{X-1}{X}\times \frac{Y-1}{Y}\times \frac{T}{T-1}\right)\; & \;\\ & \;\times \left(\frac{T}{T-2}\times \frac{T}{T-3}\right).\; & \; \end{array}$$

For *Z* = 1, we consider a pair of individuals to be the result of drawing three alleles from the base population, one of which is shared by the pair of individuals. The proportion of such pairs of individuals with IBS state *I*=1 in our data is an unbiased estimator of *P*(*I* = 1|*Z* = 1). The number of ways to select three distinct alleles from a total of *T* is *T*(*T* − 1)(*T* − 2). Among these, the genotype pairs that are *I* = 1 are the *X*(*X* − 1)*Y*, *YX*(*X* − 1), *Y*(*Y* − 1)*X*, and *XY*(*Y* − 1) that are (*AA, aa*), (*AA, aa*), (*AA, aa*), and (*AA, aa*), respectively. Thus,

$$\begin{array}{ll} \hat{P}(I=1|Z=1)=\frac{2X(X-1)Y+2\text{XY}(Y-1)}{T(T-1)(T-2)}\\ \;=\frac{2\text{XYX}}{TT^{2}}\left(\frac{X-1}{X}\times \frac{T}{T-1}\times \frac{T}{T-2}\right)\\ \;+\;\frac{2XY^{2}}{TT^{2}}\left(\frac{X-1}{X}\times \frac{T}{T-1}\times \frac{T}{T-2}\right)\\ \;=2{\hat{p}}^{2}\hat{q}\left(\frac{X-1}{X}\times \frac{T}{T-1}\times \frac{T}{T-2}\right)\\ \;+\;2\hat{p}{\hat{q}}^{2}\left(\frac{X-1}{X}\times \frac{T}{T-1}\times \frac{T}{T-2}\right). & \end{array}$$

The other conditional IBS probabilities are estimated in an analogous manner and their expressions are provided in Table one of [15].

With estimates ${\hat{P}}_{m}(I=i)$ and ${\hat{P}}_{m}(I=i|Z=z)$ for each SNP, we sum over SNPs to obtain estimates of the IBD coefficients for a given pair in the sample. Let

(3) $$\begin{array}{l} \hat{N}(I=i|Z=z)=\overset{L}{\underset{m=1}{\sum }}{\hat{P}}_{m}(I=i|Z=z)\;\;\text{and}\\ \;\hat{N}(I=i)=\overset{L}{\underset{m=1}{\sum }}{\hat{P}}_{m}(I=i), \end{array}$$

where *L* is the total number of SNPs with genotype data on both individuals. For any pair of subjects, summing equation (1) over all the SNPs and using equation (3) gives the following method-of-moment estimators of the IBD coefficients:

$$\;\begin{array}{l} \hat{P}(Z\;=\;0)\;=\;\frac{\hat{N}(I=0)}{\hat{N}(I=0|Z=0)}\\ \hat{P}(Z\;=\;1)\;=\;\frac{\hat{N}(I=1)-\hat{P}(Z=0)\times \hat{N}(I=1|Z=0)}{\hat{N}(I=1|Z=1)}\\ \hat{P}(Z\;=\;2)\;=\;\frac{\hat{N}(I\;=\;2)\;-\;\hat{P}(Z\;=\;0)\;\times \;\hat{N}(I\;=\;2|Z\;=\;0)\;-\;\hat{P}(Z=1)\;\times \;\hat{N}(I\;=\;2|Z\;=\;1)}{\hat{N}(I\;=\;2|Z\;=\;2)}\;. \end{array}$$

Adjustments to bound these estimators to values consistent with their interpretation as IBD proportions were proposed in [15]. We have not made these adjustments in our graphical displays.

### Gene drop simulation with LD

The package provides a graphical display that can be used to identify related sample pairs by plotting the estimated IBD coefficients $\hat{P}(Z=1)$ versus $\hat{P}(Z=0)$. To assess the variability of these estimators the points of the IBD plot are superposed on reference clusters obtained from one of the following relationships: unrelated, monozygotic twins/duplicates, parent-offspring, full siblings, half siblings and first cousins. These reference clusters are obtained by gene drop simulation that accounts for LD [16]. A strength of this approach is that we do not need to assume independence of marker loci. In candidate-gene association studies, this feature is important because of the dependence among a relatively small number of SNPs. Ignoring the dependence among SNPs within genes produces reference clusters that are too tight relative to the true variability, and can lead to false-positive results. We return to this point in the examples.

A graphical model is an approach to modeling the joint distribution of a set of dependent random variables when many independences or conditional independences exist between subsets of the variables. In the case of LD, it is expected that the joint distribution of alleles allong haplotypes shows such a structure. A flexible graphical model of haplotype frequencies that captures LD between loci is described in [17]. The model is fit to data from subjects that can be regarded as a population random sample; e.g., the controls in a case-control study of a rare disease. Model parameters are estimated by use of a stochastic optimization algorithm [16].

Once the LD model is fit, it is used to sample haplotypes for the founders of a pedigree. Data on the remaining members of the pedigree are simulated by gene drop. Gene drop is a method for randomly generating the genotypes of related individuals in a pedigree. Alleles are “dropped” from the founders through the pedigree according to Mendel’s laws. Multi-locus gene drop incorporates the process of recombination. To illustrate the simulation procedure, consider a parent-offspring relationship. A pedigree that encompasses this relationship is one comprised of two parents and the offspring. The founders are the parents. Parental haplotypes are simulated from the fitted LD model and are then dropped to the offspring. To mimic real data with missing genotypes, selected genotypes for a simulated individual are set to missing according to the missing genotype pattern of a randomly-sampled study subject.

Programs for fitting LD models and performing gene drop simulations are available in the Java Programs for Statistical Genetics and Computational Statistics (JPSGSC) library developed by Alun Thomas (http://balance.med.utah.edu/wiki/index.php/JPSGCS). We use the **R** package **rJPSGCS**[18] to access these programs from R.

## Results and Discussion

### Implementation

The main function in **CrypticIBDcheck** is IBDcheck(), which estimates IBD coefficients for pairs of study subjects and optionally for simulated pairs of subjects and returns an object of class IBD. The plot method for the IBD class displays the IBD coefficients for pairs of study subjects, along with prediction ellipses for known relationship pairs.

The arguments of IBDcheck() are constructed by the functions new.IBD(), filter.control() and sim.control(). The function new.IBD produces an object of class IBD suitable for input to IBDcheck(). At a minimum, such an object includes the genetic data as a snp.matrix object from the **chopsticks** package [19], a data frame of SNP information that includes chromosome and physical map positions of each SNP, and a data frame of subject information that includes a logical vector indicating whether (TRUE) or not (FALSE) each subject is to be used to estimate the conditional IBS probabilities and fit the LD model. Optionally, the SNP information may include genetic map positions, in centiMorgans. If genetic map positions are missing, they are inferred assuming the physical positions are from build 36 of the human genome. Users with SNP data on diploid non-human organisms, such as mouse or drosophila, must supply their own genetic map positions. The documentation for new.IBD() and the examples below provide further details. The function filter.control() sets options for quality control filtering of data by SNPs and by subjects, while the function sim.control() sets options that control simulation of subjects by gene drop. The respective help files and the examples below provide further details. As the fitting of LD models in IBDcheck() can be computationally demanding, users have the option of splitting computations across a **snow** cluster [20], as described in Additional file 2. The output of IBDcheck() is an object of class IBD, which includes the estimated IBD coefficients for pairs of study subjects and for simulated pairs with known relationship.

IBD objects are graphically displayed by the plot method of the class; the documentation for this method is available through help(‘‘plot.IBD''). Plots are of $\hat{P}(Z=1)$ versus $\hat{P}(Z=0)$ for pairs of study subjects, with prediction ellipses for known relationships superposed, if requested by the user. The prediction ellipses are produced from estimated IBD coefficients for a user-specified number (default 200) of simulated pairs of known relationships, assuming the distribution of estimated IBD coefficients is approximately bivariate Normal. The default setting for IBDcheck() is to omit simulated pairs from the object. When simulated pairs are omitted, plotting produces a single interactive display of estimated IBD coefficients for pairs of study subjects, on which points may be identified by clicking with the mouse. On the other hand, when the IBD object includes simulated pairs, the function returns a series of plots, which the user is prompted to view and interact with successively. The first plot to appear is non-clickable and shows the estimated IBD coefficients for all pairs of study subjects, along with the prediction ellipse for unrelated, simulated pairs. Subsequent plots are clickable and correspond to each relationship requested in the call to IBDcheck(). These relationship-specific plots are for identifying pairs of study subjects which could have the relationship. The plotting regions are restricted to the neighborhood of the prediction ellipse for the simulated pairs of that relationship, which is also drawn. If, however, the plotting region overlaps with the prediction ellipse for simulated unrelated pairs, the ellipse for simulated unrelated pairs is drawn as well. Points falling within the prediction ellipse for the relationship and outside the prediction ellipse for unrelated pairs are automatically flagged. In addition, users may click on points of study pairs that appear to be related but are not automatically flagged. The plot method produces a data frame of information on pairs that have been flagged on the different plots, either automatically or interactively by the user through clicking the mouse.

### Examples

We illustrate the features of the CrypticIBDcheck package using the genetic data Nhlsim that comes with the package. These data were simulated to mimic the characteristics of SNP genotypes in subjects of European ancestry from a candidate-gene, case-control study of non-Hodgkin Lymphoma [21]. The data set is a list comprised of (i) a snp.matrix object called snp.data with genotypes for 108 controls and 100 cases; (ii) a vector chromosome of chromosome numbers for each SNP; (iii) a vector physmap of physical map positions of each SNP, from build 36 of the human genome; and (iv) a binary vector csct with value one for cases and zero for population controls. The binary vector csct is used to select controls for fitting LD models and estimating conditional IBS probabilities. All of the information in Nhlsim is required to run IBDcheck().

We present two examples. In the first (**Default analysis with LD model fitting and gene drops**), we illustrate basic use of IBDcheck() to fit LD models and do gene drop simulations. Once the user requests simulations, there are a number of parameters, such as the types of relationships to simulate, that control the simulations. Each simulation parameter has a default value, as described in the help file for sim.control(). In the first example we use these default settings. In the second example (**Additional gene drops using previously-fit LD models**), we illustrate re-use of fitted LD models to perform additional gene drop simulations, this time for a user-specified relationship. For examples of how to use IBDcheck() to explore genome-wide data, we refer readers to the package vignette IBDcheck‐hapmap that illustrates an analysis of genome-wide data from HapMap, using thinning of markers to reduce the computational burden.

#### Default analysis with LD model fitting and gene drops

We first load the package and the Nhlsim data set.

Next we create an object of class IBD that can be used as input to the IBDcheck() function. The Nhlsim data does not include genetic map positions for the SNPs, so these will be inferred from the physical positions, assuming the physical positions are from build 36 of the human genome. We use subjects with case-control status 0 (controls) for estimating conditional IBS probabilities and fitting LD models.

In this illustration, we leave all QC filtering options (set by filter.control()) at their default values. We use sim.control() to modify the default value of simulate=FALSE to simulate=TRUE, so that reference clusters are simulated.

This call to IBDcheck() will generate 22 plain-text files in the user’s working directory that contain details of the fitted LD models for each of the 22 autosomal chromosomes. The names of these files are stored in the output IBD object:

The section **Additional gene drops using previously-fit LD models** gives an example of how to re-use these fitted LD models for performing additional gene drops. The output includes estimated IBD coefficients for pairs of subjects in the input data and for simulated pairs of subjects from the following relationships: unrelated, duplicates/MZ twins, parent-offspring, full siblings and half-siblings. Simulation of first-cousin or user-specified relationship pairs is also possible, but is not done by default. First cousins are typically not distinguishable from unrelated pairs with data from a candidate-gene association study. The estimated IBD coefficients can be plotted with the plot method for the IBD class.

**Figure 1:**
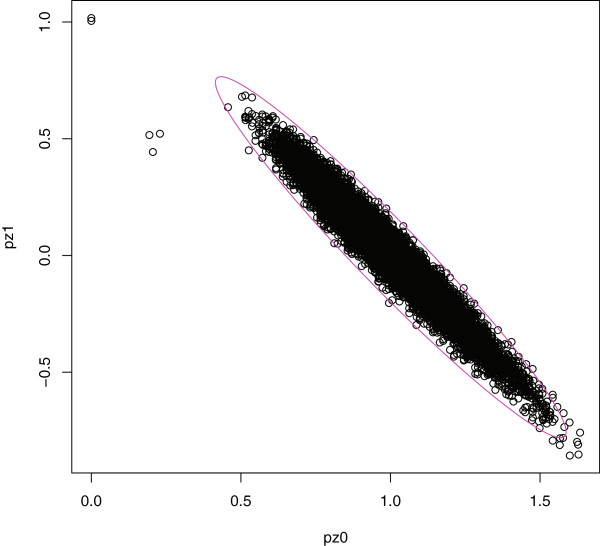
**IBD coefficients for all pairs.** Estimated IBD coefficients for all pairs of study subjects, with the prediction ellipse for unrelated pairs superposed.

**Figure 2:**
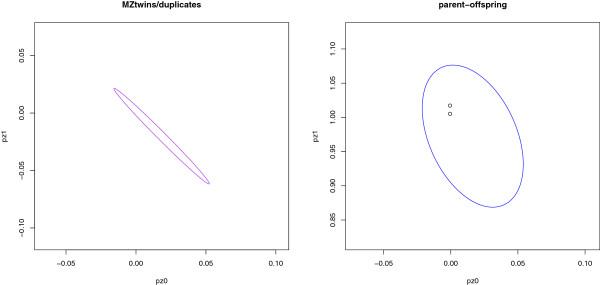
**Possible MZ twins/duplicates and parent-offspring pairs.** Observed pairs with prediction ellipses for MZ twins/duplicates (left panel) and parent-offspring pairs (right panel) superposed.

**Figure 3:**
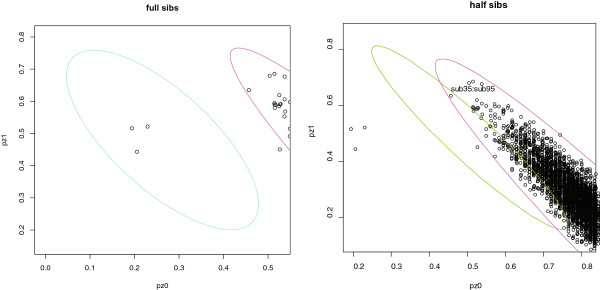
**Possible full and half sibling pairs.** Observed pairs with prediction ellipses for full-siblings pairs (left panel) and half-sibling pairs (right panel) superposed. The magenta ellipse for unrelated subjects appears on each panel.

In this example, the plotting function produces five plots, shown in Figures 1, 2, and 3, and an output data frame ibdpairs that contains information on study pairs flagged with the last four plots in Figures 2 and 3.

Figure 1 shows the non-clickable plot of the estimated IBD coefficients, *P*(*Z*=1) versus *P*(*Z*=0), for all pairs of study subjects, with the prediction ellipse for unrelated pairs superposed. The level of the prediction ellipse is left at its default value of ellipse.coverage=0.95 and, for unrelated pairs, is adjusted to account for the majority of study pairs being unrelated. Specifically, a Bonferroni-type adjustment, 1−(1−ellipse.coverage)/*n*_*p*_, is applied, where *n*_*p*_ is the number of pairs of study subjects. One purpose of the prediction ellipse is to avoid confusing the display by adding points for simulated pairs. Another purpose is to avoid having to manually click points for study pairs that appear within a cloud of points from simulated pairs. We adopted a bivariate normal approximation to the prediction ellipse because it correctly identified the majority of points in experiments with simulated data (e.g., Figure 1). However, in Figure 1, several unrelated pairs appear outside the prediction ellipse, indicating that the distribution of estimated IBD coefficients is slightly heavier-tailed than the bivariate normal approximation.

For the four other plots, shown in Figures 2 and 3, points that lie within a 95% prediction ellipse (the default level for ellipse.coverage) for the given relationship and outside the prediction ellipse for unrelated pairs are automatically flagged. In addition, these plots are clickable, and points flagged manually are added to the output dataframe. For example, on the plot for half-siblings, the point corresponding to the pair sub35 and sub95 has been manually flagged (Figure 3, right panel); this pair appears in both the prediction ellipse for unrelated pairs and the upper portion of the prediction ellipse for half siblings. Manually clicking on the point for this pair adds the following row to the output dataframe ibdpairs:

In this data set, there are no duplicate/MZ twins pairs and no pairs flagged as such (Figure 2, left panel). The two parent-offspring pairs in the Nhlsim data fall in the prediction ellipse for parent-offspring pairs (Figure 2, right panel). Similarly, all three full-sibling pairs in the Nhlsim data fall in the prediction ellipse for full siblings (Figure 3, left panel). The substantial overlap of the prediction ellipses for half siblings and unrelated pairs (Figure 3, right panel) indicates insufficient data to distinguish these two relationships. Though there are no half-sibling pairs in Nhlsim, one pair of unrelated subjects, sub31 and sub44, has atypical estimated IBD coefficients that fall within the prediction ellipse for half siblings but outside the prediction ellipse for unrelated pairs.

The unrelated pair flagged as a potential half-sibling pair is a false-positive result. We observed (results not shown) that the number of false-positive related pairs is greatly increased if we fail to take the LD between SNPs into account. Specifically, if we repeat the simulation of unrelated and half-sibling pairs of subjects assuming independent SNPs (fitLD=FALSE), we obtain 16 false-positive half sibling pairs. These observations highlight that naïvely ignoring the dependence among SNPs produces reference clusters that are too tight relative to the true variability.

#### Additional gene drops using previously-fit LD models

By far the most computationally-demanding step of IBDcheck() is the fitting of LD models. The fitted LD models are stored in plain-text files in the working directory and can be re-used for future gene drops using the argument LDfiles of sim.control(), as we now illustrate. We also demonstrate how users can create their own relationships to use as reference clusters on the IBD plot.

Setting of simulation parameters, such as the names of fitted LD model files and specification of the relationships to simulate, is done with the sim.control() function. Recall that the names of the LD files are stored in the IBD object created by a call to IBDcheck(); for example:

These fitted models are re-used by specifying their names as the argument LDfiles to sim.contol:

The sim.control() function can also be used to specify the relationships to simulate; e.g., one can obtain simulated cousin pairs with

**Figure 4:**
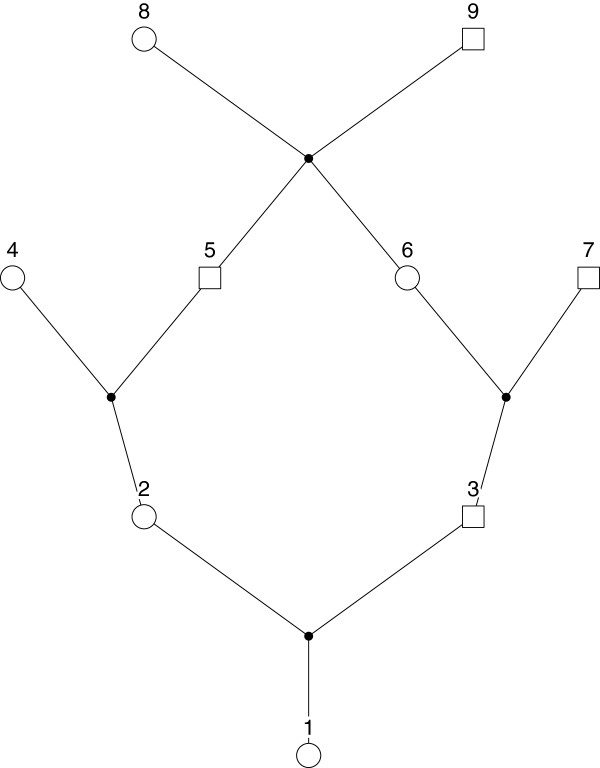
**Pedigree for an offspring of a first-cousing mariage.** Circles represent females, squares represent males. Lines of descent are indicated by connections between nodes. The mother and daughter of interest are labelled as 2 and 1, respectively.

It is also possible to obtain pairs simulated according to a user-specified relationship. In the following, the relationship of interest is parent-offspring with first cousins parents. The relationship is depicted in Figure 4, which was drawn using Pedfiddler [22]. To simulate according to this relationship, it is necessary to specify a minimal pedigree that captures the relationship between the mother and daughter and to have the mother and daughter be the first two members of the pedigree. The pedigree drawn in Figure 4 has parents (nodes 2 and 3) that are first cousins. Pedigree information is specified in a data frame whose rows describe subjects. The columns of the data frame are member IDs, the IDs of each member’s father and mother, and gender, coded as 1 for male and 2 for female. For pedigree founders, the father and mother IDs are set to zero. Specification of the pedigree in Figure 4 is as follows:

The call to IBDcheck() would then be:

where the argument filter=FALSE to filter. control() reflects the fact that there is no need to re-filter the data. On the plot of cibd.user (not shown) the prediction ellipse for simulated mother-daughter pairs where the daughter is inbred is very similar to that from simulated pairs where the daughter is not inbred (Figure 2, right panel). However, relative to the non-inbred case, the prediction ellipse in the inbred case is shifted slightly downward on the plot, reflecting the fact that the probability of 2 genes IBD is now non-zero and the probability of 1 gene IBD is therefore smaller.

## Conclusions

**CrypticIBDcheck** is an **R** package for exploring cryptic relatedness in a homogeneous sample of nominally unrelated individuals. The main function of the package, IBDcheck(), computes estimates of IBD coefficients for pairs of study subjects and, optionally, for pairs of subjects simulated to have one of several known relationships. Simulated data for a given relationship are obtained by gene drop simulation on a pedigree that captures the relationship, with founder haplotypes simulated according to an LD model fit to the data. Objects of class IBD returned by IBDcheck() are displayed by the plot method of the class. Pairs of study subjects whose estimated IBD coefficients are consistent with one of the relationships requested in the call to IBDcheck() are flagged, either automatically or interactively by user mouse-clicks, and returned in a data frame.

The methods implemented in **CrypticIBDcheck** are geared specifically towards exploring cryptic relatedness with data from candidate-gene association studies. These studies involve a relatively modest number of SNPs which are correlated because they are clustered within candidate genes. With a modest number of SNPs, the variability in the estimator of IBD coefficients cannot be ignored. Hence, reference distributions for true IBD coefficients do not adequately represent those for estimated IBD coefficients. In addition, thinning to an approximately independent and yet informative set of SNPs is not an option. Nor is ignoring LD and assuming SNPs are approximately independent. As illustrated in the Examples, ignoring LD leads to reference clusters that are too tight.

To provide guidance on the numbers of SNPs for which **CrypticIBDcheck** will be useful, we offer the following observations. A lower bound on the number of SNPs required is difficult to provide because some marker sets are more relationship-informative than others of the same size, depending on characteristics such as marker allele frequencies and the patterns of marker linkage disequilibrium (LD). In our experiments with the example data set Nhlsim, containing 1249 SNPs from 209 genes, between 325-350 SNPs from about 60 of the candidate genes appears to be adequate for identifying the parent-offspring and full sibling pairs that are present. There is no upper limit on the number of SNPs that may be used. However, the computational time for fitting LD models scales approximately linearly with the number of SNPs [16]. For the Nhlsim data, it took 40 minutes to fit the LD model. For the close relationships that we consider, genome-wide data can be reduced to a set of approximately independent SNPs with no loss of resolution. An analysis of genotypes from 16,245 approximately independent SNPs in the HapMap Luhya sample took about 2 minutes to complete. This analysis is described the package vignette IBDcheck‐HapMap.

We offer the user complete flexibility with respect to the type of relationships and number of pairs of each relationship to be simulated. Users can choose from a number of close relationships built-in to IBDcheck(), or specify their own relationships, as illustrated in the section **Additional gene drops using previously-fit LD models**.

A reviewer has pointed out that it would be useful to allow the reference distributions of IBD coefficients, represented as ellipses on the graphical displays, to be conditional on the patterns of missing genotypes in a particular pair of subjects. The intent is to allow for a two-stage analysis. In the first stage, potentially related subjects are identified using the current implementation of reference distributions, which are mixtures over the patterns of missing genotypes in all pairs of subjects. In the second stage, the reference distributions can be customized to be conditional on the missing data patterns of a pair of subjects identified in the first stage. For an assessment of whether the pair of interest has a particular relationship, distributions conditional on that pair’s missing data patterns are the most appropriate. We plan to implement an option to use a specific missing data pattern to generate the reference distributions in a future release of the package.

## Abbreviations

IBD: Identity by descent; IBD: Identity by state; LD: Linkage disequilibrium; MLE: Maximum likelihood estimator; MME: Method of moments estimator.

## Competing interests

The authors declare that they have no competing interests.

## Authors’ contributions

ANS drafted the manuscript and contributed to development of the **R** package. JG conceived of the **R** package, contributed to writing of the manuscript and contributed to development of the **R** package. BM contributed to writing of the manuscript and lead the development of the **R** package. All authors read and approved the final manuscript.

## Supplementary Material

Additional file 1 **Bias of conditional IBS estimators.** This is a PDF file that includes a calculation of the bias of the plug-in and unbiased estimators of *P*(*I*=0|*Z*=0). Bias calculations for estimators of other conditional IBS probabilities are similar. Click here for file

Additional file 2 **Splitting computations over a snow cluster.** This is a PDF file that provides details of how to split **CrypticIBDcheck** computations across a compute cluster. Click here for file
