# Supplementary material for: CrypticIBDcheck: an R package for checking cryptic relatedness in nominally unrelated individuals
Source: Source Code Biol Med. 2013 Feb 6;8:5. doi: 10.1186/1751-0473-8-5 (PMC3764977; doi:10.1186/1751-0473-8-5)
Supplement: Additional file 1 — Bias of conditional IBS estimators. This is a PDF file that includes a calculation of the bias of the plug-in and unbiased estimators of P(I=0|Z=0). Bias calculations for estimators of other conditional IBS probabilities are similar. [file 1751-0473-8-5-S1.pdf]

Appendix A: Bias of conditional IBS estimators

We calculate the bias of the plug-in and unbiased estimators of $P(I = 0|Z = 0)$. Bias calculations for estimators of other conditional IBS probabilities are similar. Throughout, consider SNPs with alleles A and a, and let p be the population allele frequency of A. Let T be twice the number of observed genotypes for the SNP in the population random sample. Let X be the number of A alleles among the T sampled and $\hat{p} = X/T$.

Bias of the plug-in estimator

We first prove the bias of the estimator $2\hat{p}^2(1-\hat{p})^2$ of $P(I = 0|Z = 0)$. Since $X \sim$ Binomial (T, p).

$$
\begin{aligned}
E[\hat{P}(I = 0|Z = 0)] &= 2E[\hat{p}^2(1-\hat{p})^2] \\
&= \frac{2}{T^4}E(X^2(T-X)^2) \\
&= \frac{2}{T^4}[T^2E(X^2) - 2TE(X^3) + E(X^4)].
\end{aligned}
$$

This calculation requires the second, third and fourth moments of the binomial distribution which can be obtained from the moment generating function:

$$
\begin{aligned}
E(X^2) &= Tp + T(T-1)p^2 \\
E(X^3) &= Tp + 3T(T-1)p^2 + T(T-1)(T-2)p^3 \\
E(X^4) &= Tp + 7T(T-1)p^2 + 6T(T-1)(T-2)p^3 + T(T-1)(T-2)(T-3)p^4.
\end{aligned}
$$

Thus,

$$
\begin{aligned}
E(2\hat{p}^2\hat{q}^2) = \frac{2}{T^4}[&Tp(T^2 - 2T + 1) + p^2T(T-1)(T^2 - 6T + 7) + \\
&p^3T(T-1)(T-2)(-2T+6) + T(T-1)(T-2)(T-3)p^4],
\end{aligned}
$$

which is not equal to $2p^2q^2$.

Unbiased estimator

We now show that the estimator of $P(I = 0|Z = 0)$ in the equation

$$
\hat{P}(I = 0|Z = 0) = \frac{2X(X-1)Y(Y-1)}{T(T-1)(T-2)(T-3)},
$$

is unbiased. Let $W = X(X-1)Y(Y-1)$ so that $E[\hat{P}(I = 0|Z = 0)] = 2E(W)/[T(T-1)(T-2)(T-3)]$. Then

$$
\begin{aligned}
E(W) &= E[(X^2 - X)(Y^2 - Y)] \\
&= E[(X^2 - X)((T-X)^2 - T + X)] \\
&= E[X^4 - 2TX^3 + X^2(T^2 + T - 1) - X(T^2 - T)]
\end{aligned}
$$

$$
\begin{aligned}
&= E(X^4) - 2TE(X^3) + (T^2 + T - 1)E(X^2) - T(T-1)E(X) \\
&= p(T - T^3 + T^2 - 2T^2 + T^3 + T^2 - T) + p^2[7T(T-1) \\
&\quad -6T^2(T-1) + T(T-1)(T^2 + T - 1)] \\
&\quad +p^3[6T(T-1)(T-2) - 2T^2(T-1)(T-2)] + T(T-1)(T-2)(T-3)p^4 \\
&= p^2[T(T-1)(T^2 - 5T + 6) + pT(T-1)(T-2)(6-2T) \\
&\quad +p^2T(T-1)(T-2)(T-3)].
\end{aligned}
$$

Therefore,

$$
\begin{aligned}
E[\hat{P}(I=0|Z=0)] &= \frac{2E(W)}{T(T-1)(T-2)(T-3)} \\
&= 2p^2\left[\frac{T^2 - 5T + 6}{(T-2)(T-3)} + 2p\frac{3-T}{T-3} + p^2\right] \\
&= 2p^2(1 - 2p + p^2) \\
&= 2p^2(1-p)^2 \\
&= 2p^2q^2.
\end{aligned}
$$
