# Supplementary material for: CrypticIBDcheck: an R package for checking cryptic relatedness in nominally unrelated individuals
Source: Source Code Biol Med. 2013 Feb 6;8:5. doi: 10.1186/1751-0473-8-5 (PMC3764977; doi:10.1186/1751-0473-8-5)
Supplement: Additional file 2 — Splitting computations over a snow cluster. This is a PDF file that provides details of how to split CrypticIBDcheck computations across a compute cluster. [file 1751-0473-8-5-S2.pdf]

Additional file 2: Splitting computations over a snow cluster

When the simulation parameters `simulate=TRUE` and `fitLD=TRUE`, the function `IBDcheck()` can be computationally demanding for data sets with more than about 1000 SNPs. For example, analysis of the `Nhlsim` data presented in the paper took about 40 minutes on an X86-based PC with a 2.4GHz, dual-core, Intel processor. Our package offers the option to use a **snow** cluster in order to split the fitting of LD models and gene drop simulations across different processors. In this appendix we distinguish between a **snow** cluster running in an interactive **R** session on a single computer, and a **snow** cluster running in batch mode on a compute cluster.

As an example of interactive use on a computer with, say, eight processors, one might create an eight-node socket cluster and use this cluster in a call to `IBDcheck()` as follows:

```
R> library("snow")
R> cl <- makeCluster(8,type="SOCK")
R> clusterEvalQ(cl,library("CrypticIBDcheck"))
R> ss <- sim.control(simulate=TRUE,cl=cl)
R> cibd.cl <- IBDcheck(dat,simparams=ss)
R> stopCluster(cl)
```

To produce the set of plots displayed in Figures 1–3 of the paper:

```
R> plot(cibd.cl)
```

The steps required to run `IBDcheck()` in batch mode on a compute cluster will depend on the setup of the compute cluster. We describe the necessary steps for a compute cluster at our institution as an example. Our local cluster uses the Torque Portable Batch System (PBS; see the documentation at `http://www.clusterresources.com/torquedocs21/usersmanual.shtml`) for running and submitting jobs and the Maui scheduler for scheduling the submitted jobs. Job submission is through a PBS file, submitted to the head node of the cluster, which in turn invokes an **R** script to carry out the computations. The following PBS file was used to fit the LD models and do gene drops with the `Nhlsim` data.

```
-------------------------Nhlsim.pbs------------------------
## submit this job to the cluster head node with
##    qsub -V Nhlsim.pbs
#!/bin/sh
#PBS -S /bin/bash
## Specify resources: 22 nodes, one processor per node, total of 16GB
## memory, estimate total time to complete of 50 minutes
#PBS -l nodes=22:ppn=1,mem=16gb,walltime=00:50:00
## Capture messages to stdout and stderr in files.
#PBS -o Nhlsimrun.out
#PBS -e Nhlsimrun.err
## Execute any shell commands needed to give the job access to R.
## On our cluster:
```

```
##     source /hpc/software/etc/colony-login
##     module load LANG/R/2.14.0
## Change to the directory from which the job was submitted
cd $PBS_O_WORKDIR
## Run the R script
R --vanilla -f Nhlsim.R
-----------------------------------------------------------
```

The PBS script calls the following **R** script to carry out the analysis. The script assumes the **R** packages **snow** and **Rmpi** have been installed on the cluster.

```
library("snow")
cl <- makeCluster(22,type="MPI")
clusterEvalQ(cl,library("CrypticIBDcheck"))
ss <- sim.control(simulate=TRUE,cl=cl)
cibd.cl <- IBDcheck(dat,simparams=ss)
save(cibd.cl,file="cibd.cl.RData")
stopCluster(cl)
```

After the `Nhlsim.pbs` job has finished, one can start an interactive **R** session in the directory that contains the file `cibd.cl.RData` and plot the results as follows:

```
R> library("CrypticIBDcheck")
R> load("cibd.cl.RData")
R> plot(cibd.cl)
```

These commands produce the set of plots displayed in Figures 1–3 of the paper.
